# Comment on “Hurdle Clearance Detection and Spatiotemporal Analysis in 400 Meters Hurdles Races Using Shoe-Mounted Magnetic and Inertial Sensor”

**DOI:** 10.3390/s20102995

**Published:** 2020-05-25

**Authors:** Marcus Schmidt, Tobias Alt, Kevin Nolte, Thomas Jaitner

**Affiliations:** 1 Institute for Sport and Sport Science, TU Dortmund University, Otto-Hahn-Straße 3, 44227 Dortmund, Germany; kevin.nolte@tu-dortmund.de (K.N.); thomas.jaitner@tu-dortmund.de (T.J.); 2 Department of Biomechanics, Performance Analysis and Strength & Conditioning, Olympic Training and Testing Centre Westphalia, 44139 Dortmund, Germany; tobias.alt@osp-westfalen.de

**Keywords:** inertial sensors, IMU, feature extraction, 400-m hurdles, spatiotemporal parameters, performance analysis

## Abstract

The recent paper “Hurdle Clearance Detection and Spatiotemporal Analysis in 400 Meters Hurdles Races Using Shoe-Mounted Magnetic and Inertial Sensor” (*Sensors* 2020, 20, 354) proposes a wearable system based on a foot-worn miniature inertial measurement unit (MIMU) and different methods to detect hurdle clearance and to identify the leading leg during 400-m hurdle races. Furthermore, the presented system identifies changes in contact time, flight time, running speed, and step frequency throughout the race. In this comment, we discuss the original paper with a focus on the ecological validity and the applicability of MIMU systems for field-based settings, such as training or competition for elite athletes.

## 1. Introduction

A successful outcome of performance analysis and coaching can be supported by useful and timely feedback to the athlete. Systematic, accurate, reliable, and valid performance monitoring can support skilled performance and promote the incorporation of scientific knowledge into coaching practice, especially in elite sports. Within this context, Falbriard, Mohr, and Aminian [1] introduce a system based on MIMU and its application in 400-m hurdles races. In applied settings, such as training interventions or competition, athletes and coaches benefit from a short feedback interval and suitable data presentation [2]. Currently, such analyses usually require time-consuming setups (e.g., multiple video cameras) and time-consuming postprocessing of the data. Recent technological developments, especially the emergence of wearable inertial sensors, provide attractive opportunities to biomechanically evaluate sports performance in a time-efficient manner outside of a laboratory. Applications of MIMU, e.g., in sprinting, prove that those systems allow for the recording of kinematic and kinetic data without spatial restrictions [3,4]. However, when operating with elite athletes in applied settings, such as training or competition, suitable systems must provide high validity and reliability to not cast uncertainty about the accuracy of the determined parameters [5]. For example, during sprinting, the contact time (CT) varies only within a few milliseconds (<10 ms) depending on the level of expertise [6] or the training period [7]. Therefore, only measurement systems with the highest accuracy can detect relevant differences. Though the validity and reliability of wearable inertial sensors seem promising, due to the small number of studies and differences in methodologies, detailed information about the algorithms applied for feature extraction and their accuracy are needed to verify the findings of applied research [3].

In sprinting, parameters like CT, flight time (FT), running speed (v), step frequency (SF), and step length (SL) represent important performance indicators, and their impact on elite performance has been investigated in several studies. However, less empirical research exists regarding spatiotemporal performance parameters and their progress during a whole 400-m or 400-m hurdles race [8,9]. This is mainly due to a lack of suitable systems to measure those parameters over the entire race, particularly for CT [10,11,12]. In this context, Falbriard, Mohr, and Aminian [1] present a concept for a wearable system for performance analysis in a field-based setting of 400-m hurdles races. The system is based on a commercially available foot-worn MIMU (Physilog4, Gait Up SA, Lausanne, Switzerland) that captures the raw data (accelerometer at 500 Hz, ±16 g; gyroscope at 500 Hz, ±2000 °/s; magnetometer at 71 Hz, ±1000 μT) and implements different methods to detect hurdle clearance and to identify the leading leg during 400-m hurdles races. Furthermore, the authors demonstrate that it is possible to identify spatiotemporal parameters throughout the race by using a temporal analysis, as in a previous validation study [13]. Therefore, we explicitly acknowledge the excellent work of the authors since it helps with increasing the scientific data about 400-m hurdles performance. The proposed system is an outstanding and promising approach for collecting sport-specific performance data in field-based settings, especially in 400-m hurdles sprinting. In the following, we will address the validity from a more applied perspective with a focus on ecological validity based on the presented findings of CT and FT. The questions we raise will hopefully contribute to a constructive discussion about the possibilities and restrictions of using MIMU sensors in applied sprinting settings.

## 2. Comments

Results of previous research in the field of 400-m sprinting show that the CTs of experienced sprinters are not below 120 ± 4 ms for the first 100 m and higher than 140 ± 5 ms for the last 100 m. At running velocities between 6.5 and 9.0 m/s during 400-m races, the maximum values of CTs are above 155 ms [10,11,12]. Compared to these values, the magnitudes of CTs presented by Falbriard, Mohr, and Aminian [1] in Table 3 seem very low. The CTs reveal mean values for all participants from 104 ± 8 ms during the first interval of the run up to 129 ± 12 ms at the end of the run. The magnitudes for all intervals of the runs are therefore far from what is expected based on values previously presented in the literature. We take this as an opportunity to discuss where these discrepancies might have emerged from.

The first possibility is that the presented magnitudes of the CT represent the correct values. However, for the mentioned experience levels of the athletes (males runtime: 57 ± 3 s; females runtime: 64 ± 3 s), as well as the running speeds (mean values for all participants ranging from 6.28 ± 0.61 up to 7.70 ± 0.64 m/s), to our knowledge, this seems unrealistic. Therefore, the maximum values of running speed that occurred in the presented sample could be of special interest. The mean values for each run interval presented in Table 3 [1] do not allow for a detailed discussion about the performance of each individual (10 males and 6 females).

Second, the estimation errors determined in the previous validation study may affect the accuracy of the CT and FT. Results of the validation study reveal an inter-trial median ± IQR bias of −15 ± 12 ms when determining the CT [13]. One, although simple, cause affecting the accuracy of the presented magnitudes might be this bias: Has this bias been implemented to correct the determined CT during the applied study in 400-m hurdles races? Assuming that the inter-trial bias can be applied to all participants of the 400-m hurdles study, an increase of CTs with a magnitude of 15 ms seems to be a reasonable correction of the results. Falbriard, Meyer, Mariani, Millet, and Aminian [13] have concluded that a speed-dependent correction should be applied to improve the accuracy of their results. Hence, such a correction would shift the results into the estimated and a more realistic range of values.

Assuming that the values measured do not represent the real performance adequately although the bias has been calculated, a deeper look into the error estimation routine might be helpful. In their validation study, the authors show that the bias and precision of the determined CTs could reach very low values. However, to evaluate the error, they computed the bias (intra-trial mean) and precision (intra-trial SD) for all steps within a trial. Afterwards, they accumulated the results from each trial and computed the median and IQR of both the bias and precision over all trials. The median and IQR functions for the inter-trial statistics were considered because the intra-trial bias and precision were not normally distributed. This already indicates that the detected errors for each individual might not sufficiently represent the bias and errors of the whole sample. Previous studies for the determination of temporal parameters during sprinting based on MIMU sensor data have shown that estimation errors underlie variations that are dependent on individual athletes [14].

**Figure 1:**
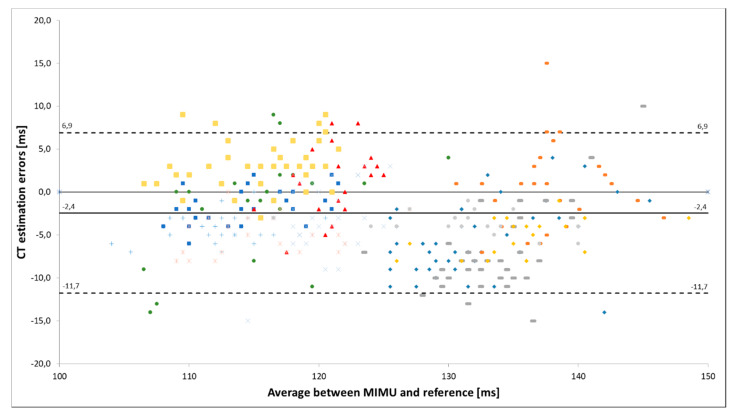
Bland–Altman plot of the estimation errors for the contact time (CT) measured by a MIMU compared to a reference system. Individual athletes are marked in different colors (modified according to Schmidt et al. [14]).

**Figure 2:**
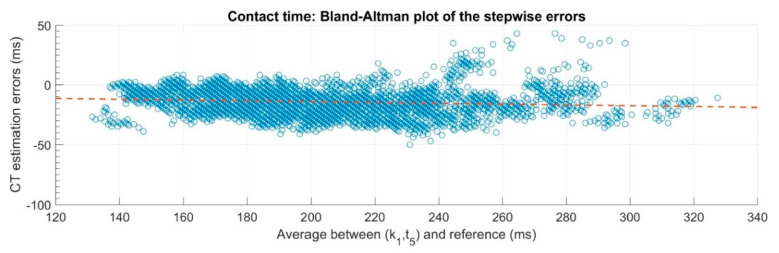
Bland–Altman plot of the estimation errors for CT of the validation study [13].

As demonstrated in Figure 1, individual performances stay within a small range of variation compared to the whole sample including different athletes. Additionally, the individual biases differ substantially between the athletes. Consequently, the error levels defined by the standard deviation of individual performances might only be applied if the bias of the individuals has been determined. If this bias is not known, the estimation error of all measures has to be considered. The Bland–Altman plots presented in the validation study (Figure 2) indicate that the estimation errors varied with the amount of CT. Furthermore, they also display a higher range of errors than the given inter-trial median ± IQR precision of less than 4 ± 3 ms. If the measurements of all subjects and steps are considered, the inter-steps random error (95% confidence interval) of 23 ms [13] should be taken into account. However, the authors gave reference to the validation study [13] when they analyzed the 400-m hurdles races but did not explain in detail how they transferred the results to their application. Therefore, we assume that they relied on the bias and precision values reported in that validation study. In this case, the application of these values on a sample of hurdles sprinters without further knowledge and consideration of their individual performance characteristics seems to be questionable. This might explain some of the unexpected findings.

Additionally, only a small number of CTs in the validation study were within the range typically expected for sprints measured during the applied setting of 400-m hurdles races (Figure 2). The validation study was done for running on an instrumented treadmill with velocities up to 20 km/h, which included CTs ranging from 132 to 354 ms [13]. However, kinematics and kinetics differ between treadmill running and sprinting compared to over-ground running and sprinting, which has been highlighted in previous research [15]. By applying the system that has been validated during running to 400-m hurdles races, the authors assumed that the results are transferable to other running conditions (surfaces, running/sprinting techniques, velocities) without any remarkable restrictions. From an ecological perspective, it would be recommended that the validation of feature extraction algorithms based on MIMU sensor data should be done within the natural sporting context to ensure that any findings are ecologically valid and meaningful. When analyzing the 400-m hurdles performance, the authors stated that the detection algorithm was affected by noise generated by the hurdle clearance movements, the adaptation steps before and after the hurdle, and the high running speeds. They addressed this aspect by including minor adaptions to the validated algorithm. These adaptions include the computation of the envelope of the signal by using wavelet transformations, and applying a high-pass (fc = 100 Hz) and low-pass filter (fc = 5 Hz). Based on a qualitative inspection, the authors assumed that the characteristics of the signal were not affected because the shape of the envelope preserved the two peaks where the initial contact and take-off occurred. However, they do not verify in detail whether the adaptions might have caused a loss of accuracy in the estimation of the CTs (e.g., as a result of time lags or smoothing). We cannot prove this in detail; however, in our experience on performance analysis in highly dynamic sports movements, the use of filters could eliminate important information of the signal shape, which might also affect the accuracy of the results.

Taken altogether, we assume that a consideration of the inter-trial bias of 15 ms, as well as the inter-steps random error of 23 ms, would shift the values of the CTs to more realistic magnitudes. This is only possible if these results can be applied to all participants of the 400-m hurdles study, meaning that all CTs are underestimated by the MIMU. However, the high range of the confidence interval would then not allow for clear conclusions about the performance exhibited within different intervals of the run or by individuals with different experience levels. Those restrictions can be overcome by calculating and applying an individual bias for each athlete. This should be done during a validation study with a suitable reference system under conditions that are comparable to the usage scenario.

## 3. Conclusions

In their study on 400-m hurdles sprinting, Falbriard, Mohr, and Aminian [1] presented CTs and FTs that differ remarkably from values known from previous research. Therefore, we discussed several aspects of data processing and error estimation, as well as individual and environmental constraints (surfaces, running/sprinting techniques, velocities). In summary, we suggest that the underlying assumptions of the error estimation, as well as the modification of the data processing algorithms, are not suitable for application in such an applied setting, and therefore these measures do not represent the real performances of athletes with a moderate level of expertise.

With our comment, we aim to highlight the specific challenges associated with the development of embedded classification systems and their application in elite sports, especially in highly dynamic sports movements, such as sprinting. This comment is explicitly not intended to impair the quality of the design and findings of Falbriard, Mohr, and Aminian [1]. Quite the opposite, we explicitly acknowledge the excellent work of the authors and thank both the *Sensors*’ editors and authors for permitting this constructive discussion about the possibilities and restrictions of MIMU sensors and the importance of focusing on the ecological validity in applied settings.
